# Mononuclear Perfluoroalkyl-Heterocyclic Complexes of Pd(II): Synthesis, Structural Characterization and Antimicrobial Activity

**DOI:** 10.3390/molecules25194487

**Published:** 2020-09-30

**Authors:** Simona Rubino, Rosa Alduina, Patrizia Cancemi, Maria Assunta Girasolo, Vita Di Stefano, Santino Orecchio, Silvestre Buscemi, Ivana Pibiri

**Affiliations:** Department of Biological, Chemical and Pharmaceutical Sciences and Technologies (STEBICEF), University of Palermo, Parco d’Orleans II, Viale delle Scienze-Bd. 16-17, 90128 Palermo, Italy; patrizia.cancemi@unipa.it (P.C.); assunta.girasolo@unipa.it (M.A.G.); vita.distefano@unipa.it (V.D.S.); santino.orecchio@unipa.it (S.O.); silvestre.buscemi@unipa.it (S.B.); ivana.pibiri@unipa.it (I.P.)

**Keywords:** mononuclear palladium complexes, perfluoroalkyl heterocyclic ligands, triazoles, antimicrobial activity, narcosis

## Abstract

Two mononuclear Pd(II) complexes [PdCl_2_(pfptp)] (**1**) and [PdCl_2_(pfhtp)] (**2**), with ligands 2-(3-perfluoropropyl-1-methyl-1,2,4-triazole-5yl)-pyridine (**pfptp**) and 2-(3-perfluoroheptyl-1-methyl-1,2,4-triazole-5yl)-pyridine (**pfhtp**), were synthesized and structurally characterized. The two complexes showed a bidentate coordination of the ligand occurring through N atom of pyridine ring and N4 atom of 1,2,4-triazole. Both complexes showed antimicrobial activity when tested against both Gram-negative and Gram-positive bacterial strains.

## 1. Introduction

In the last decade, the emergence of multi-drug resistant (MDR) bacterial strains has been considered a concern worldwide [1]. Bacterial infections in clinical and veterinary environments can frequently be due to different MDR bacterial strains, such as *Staphylococcus aureus (S. aureus)* and *Escherichia coli* (*E. coli*)*,* etc. [2,3]. In the frame of our study, we have previously synthesized, characterized and investigated the biological activity of Pt(II) complexes with 2-(5-perfluoropropyl)-1,2,4-oxadiazole-3yl)-pyridine (**pfpop**), 2-(5-perfluoroheptyl-1,2,4-oxadiazole-3yl)-pyridine (**pfhop**), 2-(3-perfluoropropyl-1-methyl-1,2,4-triazole-5yl)-pyridine (**pfptp**), and 2-(3-perfluoroheptyl-1-methyl-1,2,4-triazole-5yl)-pyridine (**pfhtp**) ligands, finding interesting data concerning both antitumor and antimicrobial activity [4,5]. Because of the severe side effects of complexes, such as cisplatin [6], in the treatment of several carcinomas, and of the cancer resistance mechanisms against the clinically utilized drug doses, in recent years, the research has been moving toward the tentative toxicity reduction of Pt(II). In order to overcome the limitations of cisplatin and its analogues [6], the application of drug delivery systems depressing toxicity and negative side effects [7], or the synthesis of new complexes with different metal ions, such as Ni(II), Pd(II), Cu(II), Ru(II), Co(II), Zn(II), and Sn(IV) [8,9,10,11,12], are some of the proposed solutions. An interesting review of the mechanistic insight on different mononuclear Pt(II) and Pt(IV) complexes and dinuclear Pt(II) complexes through spectroscopic, kinetic, and DFT measurements described the chemistry of antitumor platinum complexes, highlighting their interaction with biomolecules containing nitrogen and sulfur as donor atoms. This study could provide the basis for the development of other metal complexes with different metal ions [13].

In this context, Pd(II) complexes display structural and electronic characteristics similar to Pt(II) complexes (d_8_-electron configuration) but the high kinetic lability of Pd(II) complexes makes them inactive or highly toxic [14,15,16,17,18,19]. The choice of the ligand in the coordination chemistry of Pd(II) ion is fundamental to reduce the high cytotoxicity and to increase the solubility of palladium complexes [15]. Fluoro-substituted heterocycles are important compounds which play a role in different areas as well as agrochemicals, surfactants, medical, and pharmaceutical products [20]. The perfluoroalkyl-1,2,4-triazoles were synthesized for the first time by Brown in 1962 [21]. Later, these compounds were utilized for their potential bioactive properties [22]. Among the explored heterocyclic ligands, the triazoles, with a five membered ring and two isomer forms, 1,2,3- and 1,2,4-triazole, are worth taking into account. They are very stable to acid and basic hydrolysis in oxidizing and reducing conditions, due to their aromatic stability. Moreover, triazoles represent a very interesting class of compounds for their application in materials chemistry [23], but in particular, both 1,2,3- and 1,2,4-triazoles are widely applied in medicinal chemistry, as antitumor, anti-inflammatory, analgesic, antifungal, antibacterial, antiviral, etc. [24]. For these reasons, we have explored the antimicrobial activity of new Pd(II) complexes with 3-perfluoroalkyl-1-methyl-1,2,4-triazolyl-pyridine ligands. Both complexes were found to be active towards a Gram-negative strain, *Escherichia coli*, and a Gram-positive strain, *Kocuria rhizophila*. In addition, both complexes displayed antibacterial activity against two pathogenic *Staphylococcus aureus* Gram-positive strains.

## 2. Results and Discussion

**Scheme 1:**
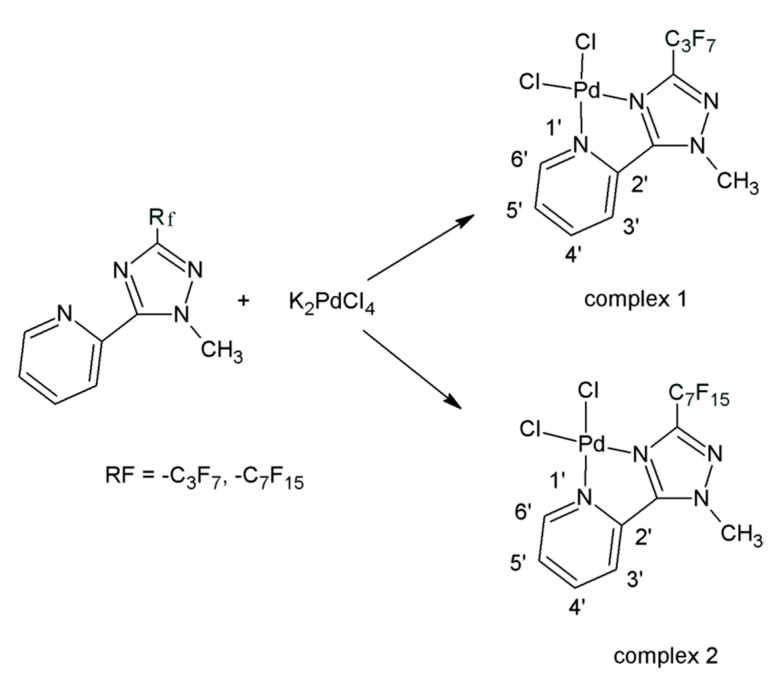
Structures of complexes **1** and **2**.

**Figure 1:**
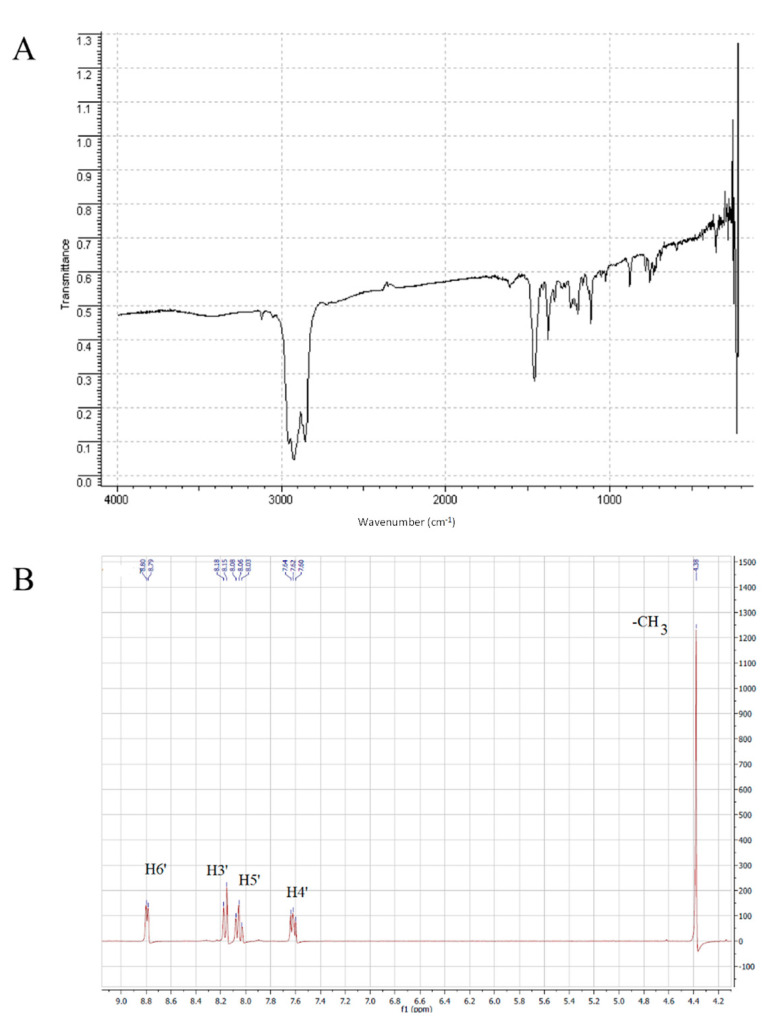
(**A**) IR spectrum and (**B**) ^1^H-NMR spectrum of complex **1.**

The IR and NMR spectra gave important information regarding the coordination modes of the ligands in the complexes (Scheme 1). In fact, the IR spectra of complexes **1** and **2** showed a slight shift of the bands due to v(CH=N) and v(C-F) vibrations of pyridine rings and perfluoroalkyl chains, after complexation of the ligands. Furthermore, the coordination of the ligand was confirmed by the stretching vibrations of Pd-N bond of **1** and **2**, at 278 and 286 cm^−1^ respectively. The symmetrical and asymmetrical stretching vibrations of Pt-Cl bonds are present at 328 and 333 cm^−1^ for complex **1** and at 344 and 328 cm^−1^ for complex **2**, respectively, indicating a square-planar arrangement of Pd(II) ion (Figure 1A, Supplementary Figure S1). The proposed structures of complexes **1** and **2** were reported in Scheme 1. The coordination mode of the complexes in solution was determined by the using of ^1^H-NMR and all assignments of resonances were based on literature data [25,26,27] and are in good agreement with the proposed structures. The numbering of protons used for ^1^H-NMR is shown in Scheme 1. For complexes **1** and **2**, the four signals of protons H6’, H5’, H3’, H4’ of pyridine rings were slightly shifted after coordination of free ligands **pfptp** and **pfhtp** (Figure 1B, Supplementary Figure S2). The bidentate coordination of the ligand occurs through the nitrogen atom of pyridine ring and N4 atom of triazole (Scheme 1).

**Figure 2:**
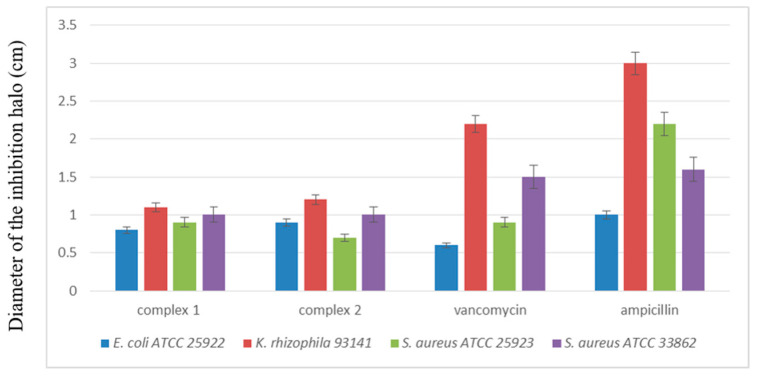
Diameter of the inhibition halo as proof of the antibacterial activity of the two complexes against *E. coli* ATCC25922, *K. rhizophila* ATCC9341, *S. aureus* ATCC 25923, *S. aureus* ATCC 33862. Vancomycin and ampicillin were used for comparison of the antibacterial activity.

The antibacterial activity of the synthesized compounds was evaluated against *Escherichia coli* ATCC25922 as an example for Gram-negative bacteria and *Kocuria rhizophila* ATCC 93411 as an example for Gram-positive bacteria by using the paper disc plate method (Figure 2). Both complexes were active against both Gram-positive and -negative bacterial strains with complex **2** featuring a better inhibition of *E. coli* growth. It is noteworthy to highlight that Gram-negative bacteria are protected by a thin protective cell wall and an additional layer of lipoproteins and lipopolysaccharides (LPS), while Gram-positive bacteria are characterized by a thick layer of cell wall and are lacking of the additional LPS membrane. An LPS layer makes the Gram-negative strains much more resistant towards both natural and synthetic molecules [5,28,29,30,31], whose entrance into the cells is hampered by the hydrophobic nature of this additional layer. Actually, the infections due to Gram-negative bacteria are more difficult to eradicate [3,32]. Thus, we assume that the perfluoroalkyl chains could allow the penetration of both compounds into Gram-negative bacteria and lead to the accumulation of these complexes into the phospholipid bilayers of the cytoplasmic membrane with the consequent alteration of the membrane properties through a process known as narcosis [31,33,34] and with the resultant cell death. This assumption is supported by the report of Wójcik et al., 2018 [35] that demonstrated the incorporation of perfluorinated compounds into model membranes resembling those typical to Gram-negative bacteria. Antibacterial activity was also tested against *S. aureus* ATCC 25923 and *S. aureus* ATCC 33862, two pathogenic, toxigenic and biofilm producer strains, frequently associated with both human and animal infections [2] and we found that both complexes were active in inhibiting the growth of both the strains with complex **1** more performing than complex **2**.

**Figure 3:**
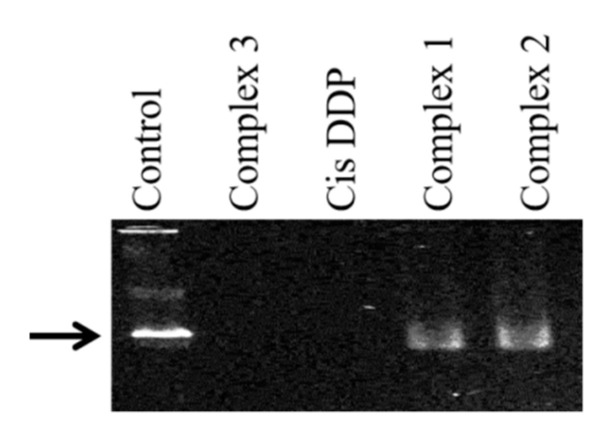
Gel electrophoresis of pUC19 plasmid DNA incubated in presence of 10 mM of complexs **1** and **2**. Plasmid DNA mobility was compared to complex **3** (Rubino et al., [5]) and cisDDP. Control indicates the plasmid DNA alone. The plasmid DNA band indicated by the arrow disappeared if DNA was bound by the compound.

**Scheme 2:**
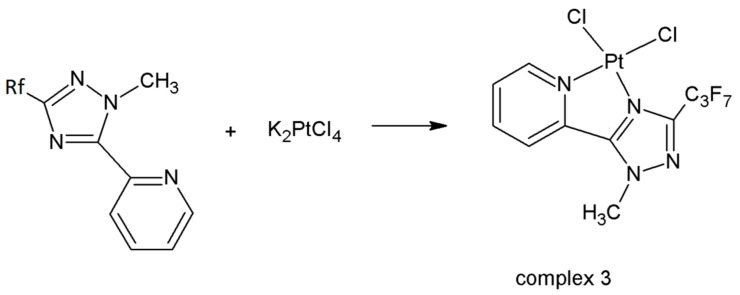
Structure of complex **3**.

In addition, we evaluated whether complexes **1** and **2** could explicate their mode of action by binding to DNA molecules, as cisDDP and its derivatives are reported to do [5,15]. Mobility shift assay of a model DNA molecule (plasmid pUC19) demonstrated that both complexes did not bind to DNA (Figure 3), differently from the complex **3**, previously prepared and characterized [5] containing the short perfluorinated chain that strongly binds to plasmid DNA. The structure of the complex **3** was reported in Scheme 2.

## 3. Materials and Methods 

### 3.1. Instrumentation

Reagents and solvents were used as received. Ligands were synthesized as described in literature [4]. Synthesis of complexes was executed with the exclusion of direct light. Elemental analysis for C, H, N was performed at the Laboratorio di Microanalisi (University of Padova, Italy). IR spectra (Nujol) were registered with a Shimadzu FTIR-8300 instrument (Shimadzu Scientific Instruments Inc., Maryland, USA). ^1^H spectra were recorded on a Bruker 300 Advance spectrometer (Bruker, Massachusetts, USA), operating at 300 MHz. All complexes were dissolved in DMSO-*d*_6_. The chemical shifts were expressed as δ (ppm) with tetramethylsilane (TMS) as an internal standard for ^1^H-NMR. Chlorine was determined by potentiometric titration with standard silver nitrate after combustion in pure oxygen according to Schöniger [36]. The percentage of palladium was measured by absorption spectroscopy using a Perkin-Elmer 372 atomic absorption spectrometer (Perkin-Elmer, Milano, Italy) according to the appropriate standards. The molar conductivities were determined in DMSO at 10^−3^ M at 25 °C with a Crison GLP 31 Model Conductometer (Mettler Toledo, Ohio, USA). Mass spectrometry (MS) spectra of the complexes were determined with Q-Exactive High Resolution mass spectrometer (Thermo Fischer Scientific, Munchen, Germany equipped with the HESI electrospray source. Melting points were detected on a Kofler plate with Reichart-Thermovar hotstage apparatus (Reichart Thermovar) Both complexes presented a low solubility even in DMSO-*d*_6_, thus preventing the acquisition of useful ^13^C-NMR spectra.

### 3.2. Biological Sample and Reagents

The paper disc plate method was used to determine the antimicrobial activity of the complexes **1** and **2** as previously described [37]. The antimicrobial activity was investigated against the Gram-negative *Escherichia coli* ATCC 25922 and the Gram-positive *Kocuria rhizophila* ATCC93411, *Staphylococcus aureus* ATCC 25923, and *S. aureus* ATCC 33862. Bacterial suspensions of each microorganism were prepared in LB medium with a total bacterial count of approximately 10^8^ cells/mL. A 100 µL aliquot of bacterial suspension was spread onto LB-agar plates. Since compounds were dissolved in DMSO, a pure solution of DMSO and sterile distilled water were used to soak paper disks that were used as negative controls. The same amount (5 µM) of the complexes were directly spotted on sterile Whatman filter paper discs that were put on the overlay of each bacterial suspension on different LB-agar plates. After overnight incubation at 37 °C, growth inhibition halos were observed and compared to those obtained using 5 μM of vancomycin and ampicillin. The antimicrobial activity was calculated at least as a mean of three replicates. DNA binding activity of 10 mM of the complexes was evaluated by using electrophoretic mobility shift assays (EMSA) of 100 ng of pUC19 DNA plasmid as described elsewhere [38]. At the end of the electrophoresis, the gel was ethidium bromide stained and photographed.

### 3.3. Synthesis of Complex ***1***

Complex **1** was synthesized from an aqueous solution of K_2_PdCl_4_ (0.149 g, 4.6 × 10^−4^ mol) (10 mL) that was added dropwise to an ethanolic solution (30 mL) of pfptp (0.151 g, 4.6 × 10^−4^ mol), with stirring at 50 °C for 1 h and at room temperature for 24 h. Methods of synthesis of the ligands were reported in the literature [4,39,40,41,42]. The solid was filtered, washed with water, ethanol and dried in vacuo over P_4_O_10_. K_2_PdCl_4_ was prepared dissolving PdCl_2_ (0.177 g) in an aqueous solution of KCl (0.149 g). The solution was stirred for half an hour at room temperature. Yield 70%. Anal. Calc. for (C_11_H_7_N_4_F_7_PdCl_2_): C, 26.14; H, 1.40; N, 11.08; Cl, 14.03; Pd, 21.05%. Found: C, 26.00; H, 1.30; N, 11.05; Cl, 13.70; Pd, 21.45%. Melting point: >250 °C (decomp.). Λ_M_ =11 µS (indicative of neutral complex) [39]. IR (cm^−1^) for free ligand: 2853 *v*(C-H), 1590 *v*(CH=N), 1241-1147 *v*(C-F), and for the complex: 2853 *v* (C-H), 1611 *v*(CH=N), 1238-1196 *v*(C-F), 333 and 328 *v*(Pd-Cl), 278 *v*(Pd-N) Figure 1A). ^1^H-NMR of the ligands (DMSO-*d*_6_, 300 MHz) δ ppm were reported in literature [4,20,21]. For the complex (DMSO-*d*_6_, 300 MHz) δ ppm: 8.81 (d, 1H, C6’H), 8.16 (d, 1H, C3’H), 8.04 (t, 1H, C5’H), 7.63 (t, 1H, C4’H), 4.39 (s, 3H, -CH_3_). ^3^*J*_C3HC4H_ = 8 Hz; ^3^*J*_C6HC5H_ = 3 Hz (Figure 1B). ESI(+)-MS: [M − Cl]^+^ [C_11_H_7_F_7_N_4_ClPd] calculated: 468.92768, found: 468.92405 *m*/*z*, Supplementary Figure S3.

### 3.4. Synthesis of Complex ***2***

Complex **2** was prepared following the same procedure for complex **1** by an aqueous solution of K_2_PdCl_4_ (0.149 g, 4.6 × 10^−4^ mol) (10 mL) that was added dropwise to an ethanolic solution (30 mL) of pfhtp (0.243 g, 4.6 × 10^−4^ mol). Yield 68%. Anal. Calc. for (C_15_H_7_N_4_F_15_PdCl_2_): C, 25.54; H, 1.00; N, 7.94; Cl, 10.05; Pd, 15.08%. Found: C, 25.14; H, 1.00; N, 7.65; Cl, 9.96; Pd, 14.70%. Melting point: >250 °C (decomp.). Λ_M_ = 8 µS (indicative of neutral complex) [42]. IR (cm^−1^) for free ligand: 2853 *v* (C-H), 1590 *v* (CH=N), 1247-1147 *v*(C-F), and for the complex 2853 *v* (C-H), 1598 *v*(CH=N), 1274-1147 *v*(C-F), 344 and 328 *v*(Pd-Cl) and 286, 277 *v*(Pd-N), Supplementary Figure S1. ^1^H-NMR for the complex (DMSO, 300 MHz) δ ppm: 8.80 (d, 1H, C6’H), 8.16 (d, 1H, C3’H), 8.06 (t, 1H, C5’H), 7.63 (t, 1H, C4’H), 4.38 (s, 3H, -CH_3_); ^3^*J*_C3HC4H_ = 8 Hz; ^3^*J*_C6HC5H_ = 6.0 Hz Fig. S2. ESI(+)-MS: [M − Cl]^+^ [C_15_H_7_N_4_ClF_15_Pd] calculated: 668.91491, found: 668.91565 *m*/*z*, Supplementary Figure S4.

## 4. Conclusions

The experience of Pt(II) complexes with perfluoroalkyl heterocyclic ligands, their synthesis, and biological studies (anti-proliferative and antimicrobial activity) led us to investigate the synthesis and the antibacterial activity of new complexes constituted by 2-(3-perfluoropropyl-1-methyl-1,2,4-triazole-5yl)-pyridine (**pfptp**) or 2-(3-perfluoroheptyl-1-methyl-1,2,4-triazole-5yl)-pyridine (**pfhtp**) as ligands and Pd(II), as metal ion, in replacement of Pt(II) ion. As already observed for Pt(II) complexes, in Pd(II) complexes, the coordination occurs through the nitrogen atom of pyridine rig and N4 atom of triazole, the geometry of metal is square-planar. Complexes **1** and **2** showed activity against the Gram-negative *E. coli* ATCC 25922 and the Gram-positive *K. rhizophila* ATCC 93411, *S. aureus* ATCC 25923, and *S. aureus* ATCC 33862 strains. We assume that the activity of both complexes might be dependent upon the perfluoroalkyl chains that could allow their penetration into the bacterial membrane.

## Supplementary Materials

The following are available online, Figure S1: FTIR spectra of complex (**2**), Figure S2: ^1^H-NMR of complex (**2**) in DMSO-*d*_6_ solvent, Figure S3: ESI(+)-(MS) [M − Cl]^+^ spectra of complex (**1**), Figure S4: ESI(+)-(MS) [M − Cl]^+^ spectra of complex (**2**).
