# Supplementary material for: Mononuclear Perfluoroalkyl-Heterocyclic Complexes of Pd(II): Synthesis, Structural Characterization and Antimicrobial Activity
Source: Molecules. 2020 Sep 30;25(19):4487. doi: 10.3390/molecules25194487 (PMC7582383; doi:10.3390/molecules25194487)
Supplement: Supplementary file 1 [file molecules-25-04487-s001.pdf]

Supporting Information

Mononuclear perfluoroalkyl-heterocyclic complexes of Pd(II): synthesis, structural characterization and antimicrobial activity

Simona Rubino[1*], Rosa Alduina[1*], Patrizia Cancemi[1], Maria Assunta Girasolo[1], Vita Di Stefano[1], Santino Orecchio[1], Silvestre Buscemi[1], Ivana Pibiri[1]

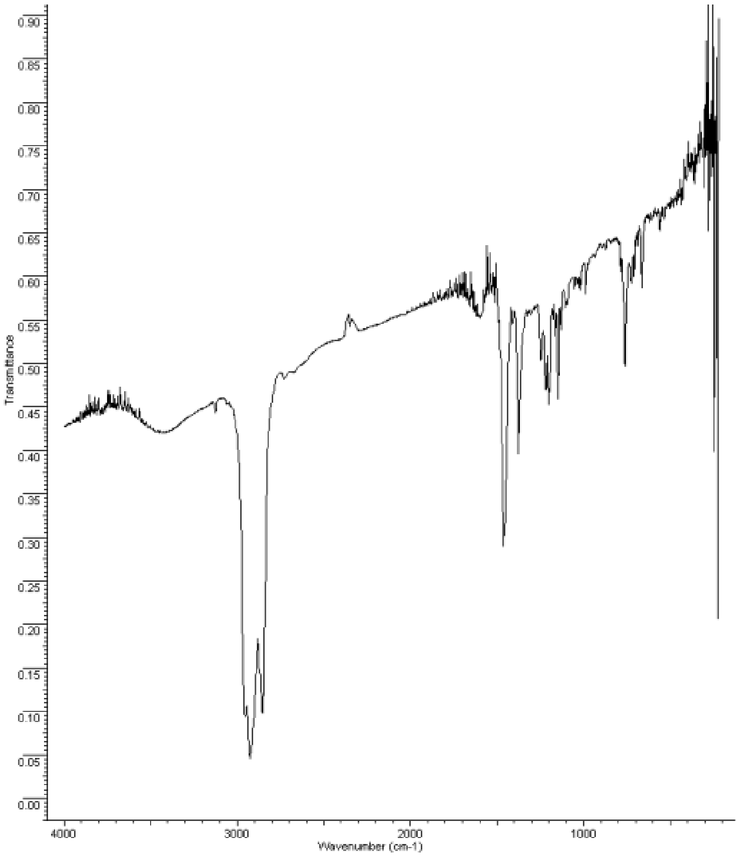

Figure S1. FTIR spectra of complex (2).

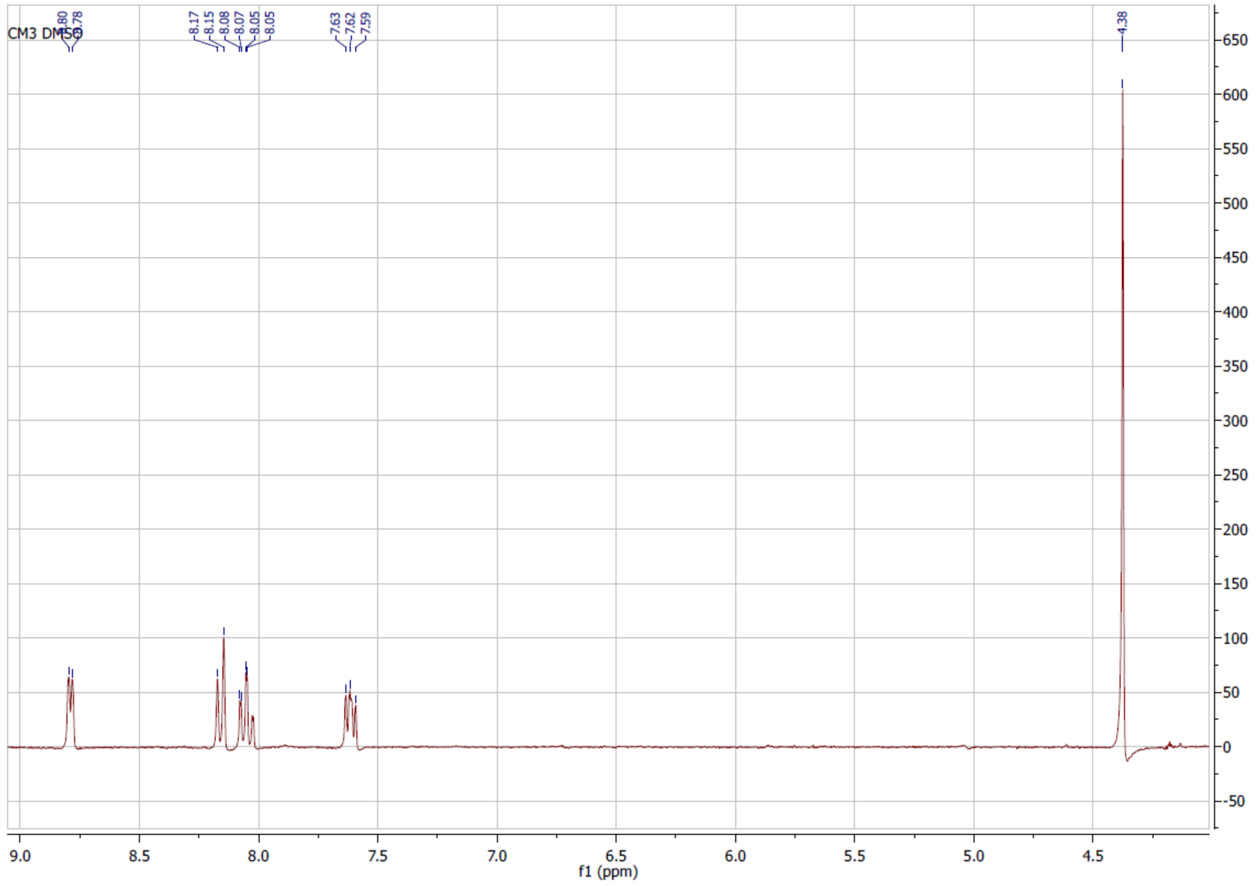

Figure S2. ^{1}H NMR of complex (2) in DMSO-d_6 solvent.

CM6:

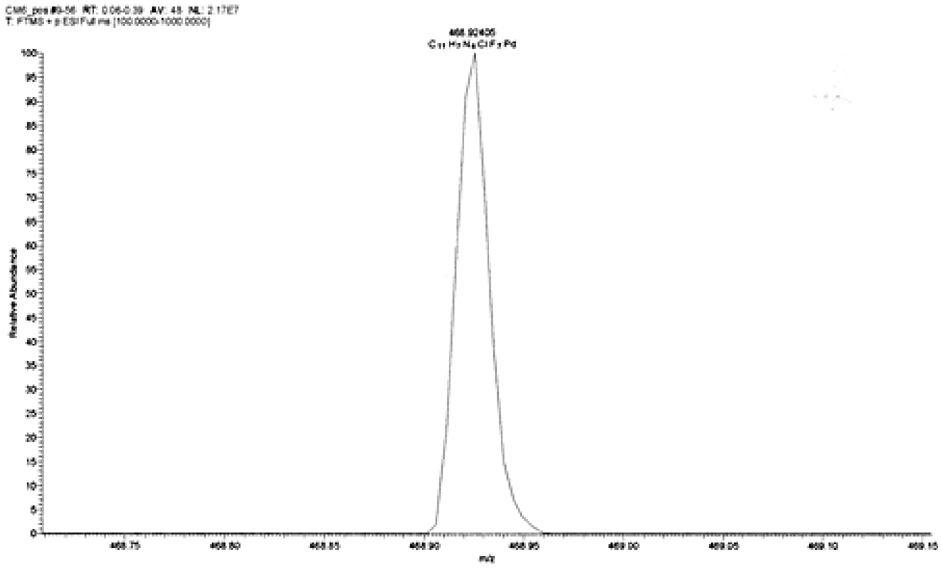

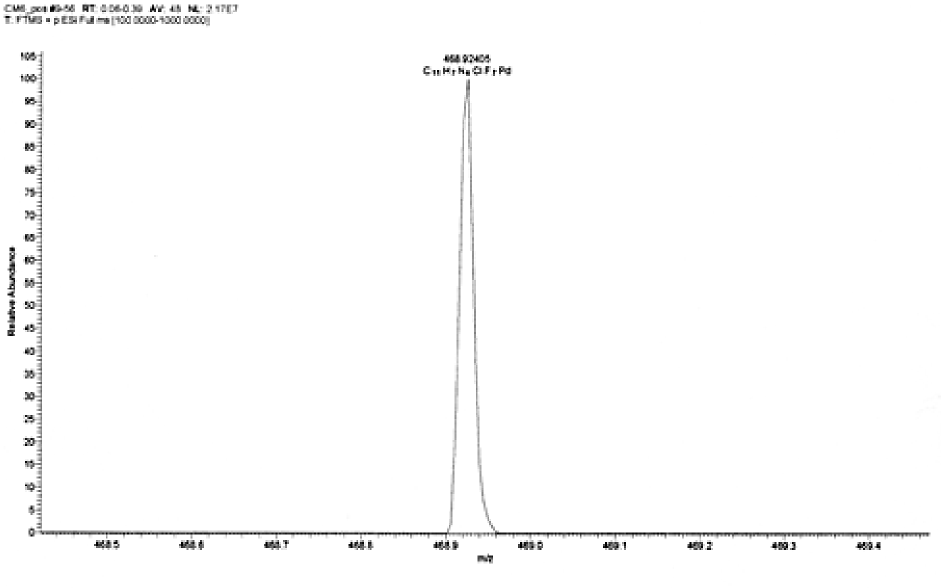

Figure S3. ESI(+)-(MS) $[M\text{-}Cl]^+$ spectra of complex (1)

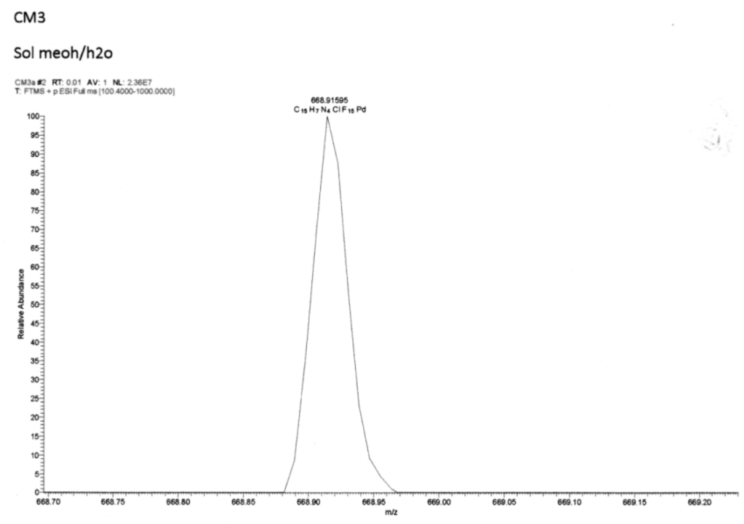

Figure S4. ESI(+)-(MS) $[M\text{-}Cl]^+$ spectra of complex (2)
